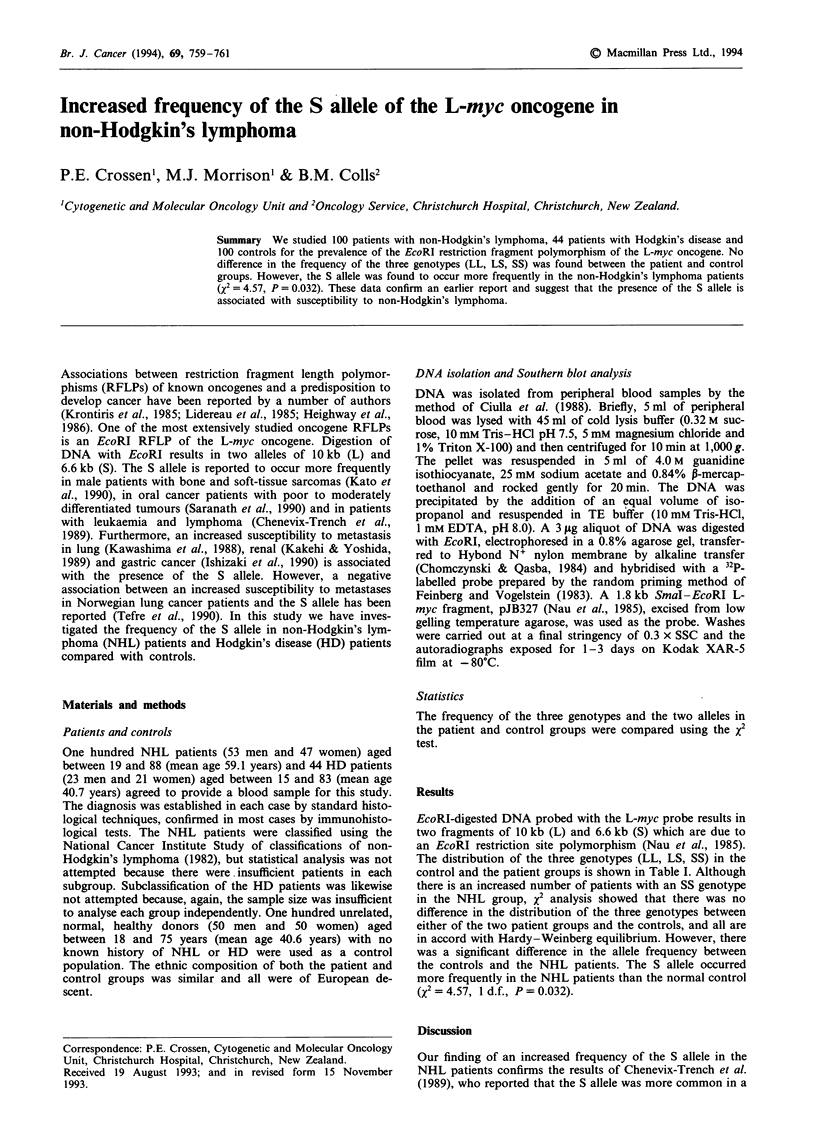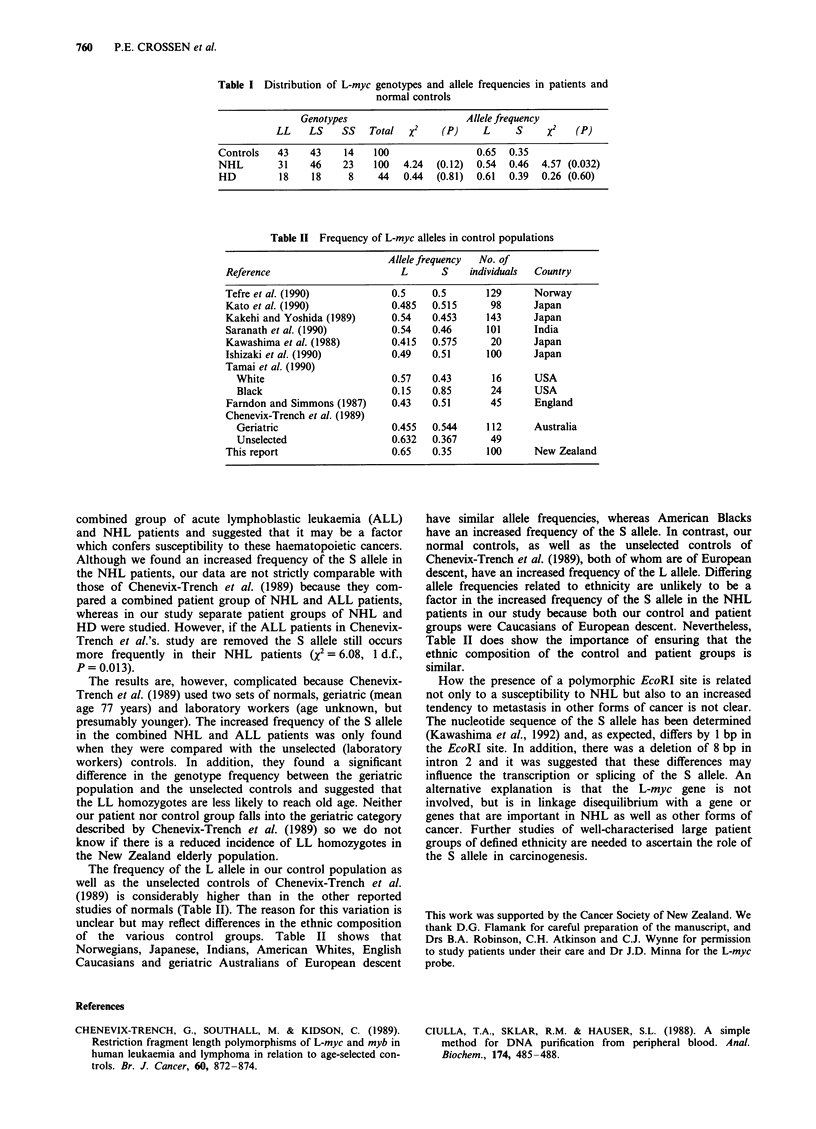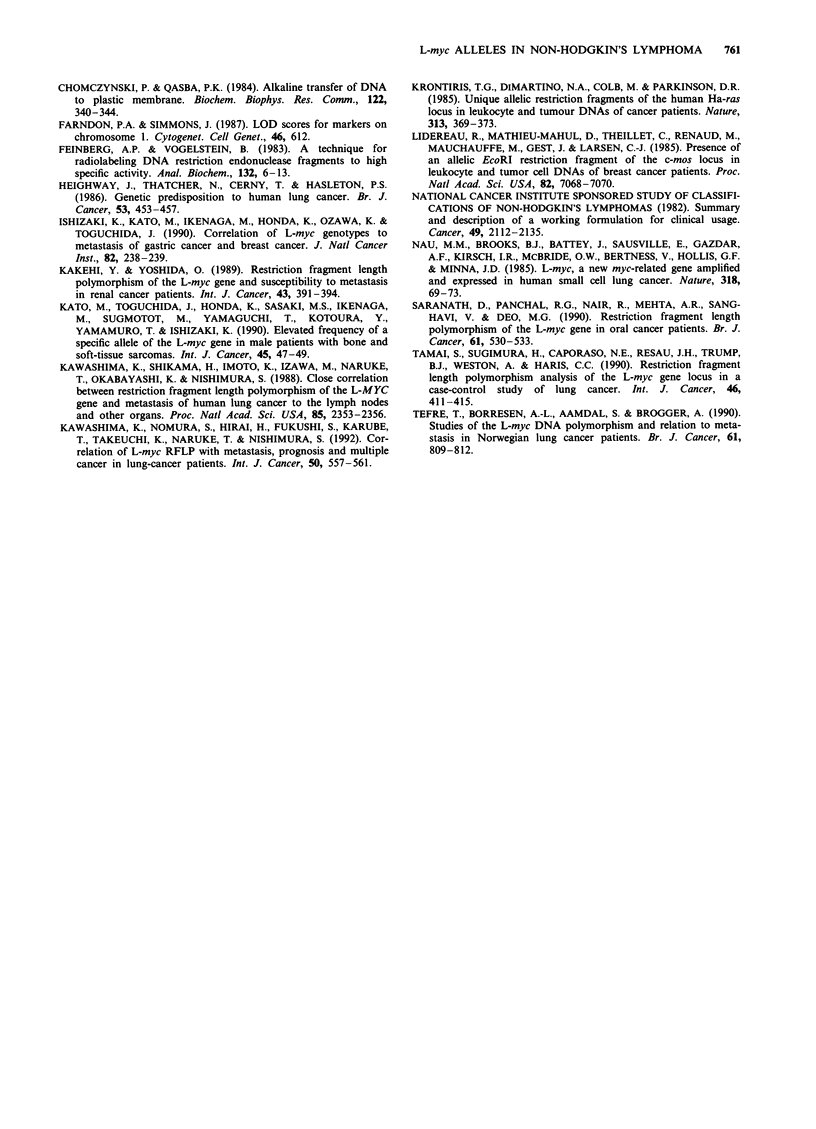# Increased frequency of the S allele of the L-myc oncogene in non-Hodgkin's lymphoma.

**DOI:** 10.1038/bjc.1994.143

**Published:** 1994-04

**Authors:** P. E. Crossen, M. J. Morrison, B. M. Colls

**Affiliations:** Cytogenetic and Molecular Oncology Unit, Christchurch Hospital, New Zealand.

## Abstract

We studied 100 patients with non-Hodgkin's lymphoma, 44 patients with Hodgkin's disease and 100 controls for the prevalence of the EcoRI restriction fragment polymorphism of the L-myc oncogene. No difference in the frequency of the three genotypes (LL, LS, SS) was found between the patient and control groups. However, the S allele was found to occur more frequently in the non-Hodgkin's lymphoma patients (chi 2 = 4.57, P = 0.032). These data confirm an earlier report and suggest that the presence of the S allele is associated with susceptibility to non-Hodgkin's lymphoma.


Br. J. Cancer (1994), 69, 759-761 © Macmillan Press Ltd., 1994

### Increased frequency of the S allele of the L-*myc* oncogene in non-Hodgkin's lymphoma

P.E. Crossen[1], M.J. Morrison[1] & B.M. Colls[2]

[1]*Cytogenetic and Molecular Oncology Unit and* [2]*Oncology Service, Christchurch Hospital, Christchurch, New Zealand.*

**Summary** We studied 100 patients with non-Hodgkin's lymphoma, 44 patients with Hodgkin's disease and 100 controls for the prevalence of the *Eco*RI restriction fragment polymorphism of the L-*myc* oncogene. No difference in the frequency of the three genotypes (LL, LS, SS) was found between the patient and control groups. However, the S allele was found to occur more frequently in the non-Hodgkin's lymphoma patients ($\chi^2 = 4.57$, $P = 0.032$). These data confirm an earlier report and suggest that the presence of the S allele is associated with susceptibility to non-Hodgkin's lymphoma.

Associations between restriction fragment length polymorphisms (RFLPs) of known oncogenes and a predisposition to develop cancer have been reported by a number of authors (Krontiris *et al.*, 1985; Lidereau *et al.*, 1985; Heighway *et al.*, 1986). One of the most extensively studied oncogene RFLPs is an *Eco*RI RFLP of the L-*myc* oncogene. Digestion of DNA with *Eco*RI results in two alleles of 10 kb (L) and 6.6 kb (S). The S allele is reported to occur more frequently in male patients with bone and soft-tissue sarcomas (Kato *et al.*, 1990), in oral cancer patients with poor to moderately differentiated tumours (Saranath *et al.*, 1990) and in patients with leukaemia and lymphoma (Chenevix-Trench *et al.*, 1989). Furthermore, an increased susceptibility to metastasis in lung (Kawashima *et al.*, 1988), renal (Kakehi & Yoshida, 1989) and gastric cancer (Ishizaki *et al.*, 1990) is associated with the presence of the S allele. However, a negative association between an increased susceptibility to metastases in Norwegian lung cancer patients and the S allele has been reported (Tefre *et al.*, 1990). In this study we have investigated the frequency of the S allele in non-Hodgkin's lymphoma (NHL) patients and Hodgkin's disease (HD) patients compared with controls.

### Materials and methods

### *Patients and controls*

One hundred NHL patients (53 men and 47 women) aged between 19 and 88 (mean age 59.1 years) and 44 HD patients (23 men and 21 women) aged between 15 and 83 (mean age 40.7 years) agreed to provide a blood sample for this study. The diagnosis was established in each case by standard histological techniques, confirmed in most cases by immunohistological tests. The NHL patients were classified using the National Cancer Institute Study of classifications of non-Hodgkin's lymphoma (1982), but statistical analysis was not attempted because there were insufficient patients in each subgroup. Subclassification of the HD patients was likewise not attempted because, again, the sample size was insufficient to analyse each group independently. One hundred unrelated, normal, healthy donors (50 men and 50 women) aged between 18 and 75 years (mean age 40.6 years) with no known history of NHL or HD were used as a control population. The ethnic composition of both the patient and control groups was similar and all were of European descent.

Correspondence: P.E. Crossen, Cytogenetic and Molecular Oncology Unit, Christchurch Hospital, Christchurch, New Zealand.
Received 19 August 1993; and in revised form 15 November 1993.

### *DNA isolation and Southern blot analysis*

DNA was isolated from peripheral blood samples by the method of Ciulla *et al.* (1988). Briefly, 5 ml of peripheral blood was lysed with 45 ml of cold lysis buffer (0.32 M sucrose, 10 mM Tris-HCl pH 7.5, 5 mM magnesium chloride and 1% Triton X-100) and then centrifuged for 10 min at 1,000 *g*. The pellet was resuspended in 5 ml of 4.0 M guanidine isothiocyanate, 25 mM sodium acetate and 0.84% β-mercaptoethanol and rocked gently for 20 min. The DNA was precipitated by the addition of an equal volume of isopropanol and resuspended in TE buffer (10 mM Tris-HCl, 1 mM EDTA, pH 8.0). A 3 μg aliquot of DNA was digested with *Eco*RI, electrophoresed in a 0.8% agarose gel, transferred to Hybond $N^+$ nylon membrane by alkaline transfer (Chomczynski & Qasba, 1984) and hybridised with a $^{32}P$-labelled probe prepared by the random priming method of Feinberg and Vogelstein (1983). A 1.8 kb *Sma*I-*Eco*RI L-*myc* fragment, pJB327 (Nau *et al.*, 1985), excised from low gelling temperature agarose, was used as the probe. Washes were carried out at a final stringency of 0.3 × SSC and the autoradiographs exposed for 1-3 days on Kodak XAR-5 film at −80°C.

### *Statistics*

The frequency of the three genotypes and the two alleles in the patient and control groups were compared using the $\chi^2$ test.

### Results

*Eco*RI-digested DNA probed with the L-*myc* probe results in two fragments of 10 kb (L) and 6.6 kb (S) which are due to an *Eco*RI restriction site polymorphism (Nau *et al.*, 1985). The distribution of the three genotypes (LL, LS, SS) in the control and the patient groups is shown in Table I. Although there is an increased number of patients with an SS genotype in the NHL group, $\chi^2$ analysis showed that there was no difference in the distribution of the three genotypes between either of the two patient groups and the controls, and all are in accord with Hardy-Weinberg equilibrium. However, there was a significant difference in the allele frequency between the controls and the NHL patients. The S allele occurred more frequently in the NHL patients than the normal control ($\chi^2 = 4.57$, 1 d.f., $P = 0.032$).

### Discussion

Our finding of an increased frequency of the S allele in the NHL patients confirms the results of Chenevix-Trench *et al.* (1989), who reported that the S allele was more common in a

**Table I** Distribution of L-*myc* genotypes and allele frequencies in patients and normal controls

| | *Genotypes* | | | | | | *Allele frequency* | | | |
|---|---|---|---|---|---|---|---|---|---|---|
| | *LL* | *LS* | *SS* | *Total* | $\chi^2$ | *(P)* | *L* | *S* | $\chi^2$ | *(P)* |
| Controls | 43 | 43 | 14 | 100 | | | 0.65 | 0.35 | | |
| NHL | 31 | 46 | 23 | 100 | 4.24 | (0.12) | 0.54 | 0.46 | 4.57 | (0.032) |
| HD | 18 | 18 | 8 | 44 | 0.44 | (0.81) | 0.61 | 0.39 | 0.26 | (0.60) |

**Table II** Frequency of L-*myc* alleles in control populations

| *Reference* | *Allele frequency L* | *S* | *No. of individuals* | *Country* |
|---|---|---|---|---|
| Tefre *et al.* (1990) | 0.5 | 0.5 | 129 | Norway |
| Kato *et al.* (1990) | 0.485 | 0.515 | 98 | Japan |
| Kakehi and Yoshida (1989) | 0.54 | 0.453 | 143 | Japan |
| Saranath *et al.* (1990) | 0.54 | 0.46 | 101 | India |
| Kawashima *et al.* (1988) | 0.415 | 0.575 | 20 | Japan |
| Ishizaki *et al.* (1990) | 0.49 | 0.51 | 100 | Japan |
| Tamai *et al.* (1990) | | | | |
| White | 0.57 | 0.43 | 16 | USA |
| Black | 0.15 | 0.85 | 24 | USA |
| Farndon and Simmons (1987) | 0.43 | 0.51 | 45 | England |
| Chenevix-Trench *et al.* (1989) | | | | |
| Geriatric | 0.455 | 0.544 | 112 | Australia |
| Unselected | 0.632 | 0.367 | 49 | |
| This report | 0.65 | 0.35 | 100 | New Zealand |

combined group of acute lymphoblastic leukaemia (ALL) and NHL patients and suggested that it may be a factor which confers susceptibility to these haematopoietic cancers. Although we found an increased frequency of the S allele in the NHL patients, our data are not strictly comparable with those of Chenevix-Trench *et al.* (1989) because they compared a combined patient group of NHL and ALL patients, whereas in our study separate patient groups of NHL and HD were studied. However, if the ALL patients in Chenevix-Trench *et al.*'s. study are removed the S allele still occurs more frequently in their NHL patients ($\chi^2 = 6.08$, 1 d.f., $P = 0.013$).

The results are, however, complicated because Chenevix-Trench *et al.* (1989) used two sets of normals, geriatric (mean age 77 years) and laboratory workers (age unknown, but presumably younger). The increased frequency of the S allele in the combined NHL and ALL patients was only found when they were compared with the unselected (laboratory workers) controls. In addition, they found a significant difference in the genotype frequency between the geriatric population and the unselected controls and suggested that the LL homozygotes are less likely to reach old age. Neither our patient nor control group falls into the geriatric category described by Chenevix-Trench *et al.* (1989) so we do not know if there is a reduced incidence of LL homozygotes in the New Zealand elderly population.

The frequency of the L allele in our control population as well as the unselected controls of Chenevix-Trench *et al.* (1989) is considerably higher than in the other reported studies of normals (Table II). The reason for this variation is unclear but may reflect differences in the ethnic composition of the various control groups. Table II shows that Norwegians, Japanese, Indians, American Whites, English Caucasians and geriatric Australians of European descent have similar allele frequencies, whereas American Blacks have an increased frequency of the S allele. In contrast, our normal controls, as well as the unselected controls of Chenevix-Trench *et al.* (1989), both of whom are of European descent, have an increased frequency of the L allele. Differing allele frequencies related to ethnicity are unlikely to be a factor in the increased frequency of the S allele in the NHL patients in our study because both our control and patient groups were Caucasians of European descent. Nevertheless, Table II does show the importance of ensuring that the ethnic composition of the control and patient groups is similar.

How the presence of a polymorphic *Eco*RI site is related not only to a susceptibility to NHL but also to an increased tendency to metastasis in other forms of cancer is not clear. The nucleotide sequence of the S allele has been determined (Kawashima *et al.*, 1992) and, as expected, differs by 1 bp in the *Eco*RI site. In addition, there was a deletion of 8 bp in intron 2 and it was suggested that these differences may influence the transcription or splicing of the S allele. An alternative explanation is that the L-*myc* gene is not involved, but is in linkage disequilibrium with a gene or genes that are important in NHL as well as other forms of cancer. Further studies of well-characterised large patient groups of defined ethnicity are needed to ascertain the role of the S allele in carcinogenesis.

This work was supported by the Cancer Society of New Zealand. We thank D.G. Flamank for careful preparation of the manuscript, and Drs B.A. Robinson, C.H. Atkinson and C.J. Wynne for permission to study patients under their care and Dr J.D. Minna for the L-*myc* probe.

### References

CHENEVIX-TRENCH, G., SOUTHALL, M. & KIDSON, C. (1989). Restriction fragment length polymorphisms of L-*myc* and *myb* in human leukaemia and lymphoma in relation to age-selected controls. *Br. J. Cancer*, **60**, 872–874.

CIULLA, T.A., SKLAR, R.M. & HAUSER, S.L. (1988). A simple method for DNA purification from peripheral blood. *Anal. Biochem.*, **174**, 485–488.

CHOMCZYNSKI, P. & QASBA, P.K. (1984). Alkaline transfer of DNA to plastic membrane. *Biochem. Biophys. Res. Comm.*, **122**, 340–344.

FARNDON, P.A. & SIMMONS, J. (1987). LOD scores for markers on chromosome 1. *Cytogenet. Cell Genet.*, **46**, 612.

FEINBERG, A.P. & VOGELSTEIN, B. (1983). A technique for radiolabeling DNA restriction endonuclease fragments to high specific activity. *Anal. Biochem.*, **132**, 6–13.

HEIGHWAY, J., THATCHER, N., CERNY, T. & HASLETON, P.S. (1986). Genetic predisposition to human lung cancer. *Br. J. Cancer*, **53**, 453–457.

ISHIZAKI, K., KATO, M., IKENAGA, M., HONDA, K., OZAWA, K. & TOGUCHIDA, J. (1990). Correlation of L-*myc* genotypes to metastasis of gastric cancer and breast cancer. *J. Natl Cancer Inst.*, **82**, 238–239.

KAKEHI, Y. & YOSHIDA, O. (1989). Restriction fragment length polymorphism of the L-*myc* gene and susceptibility to metastasis in renal cancer patients. *Int. J. Cancer*, **43**, 391–394.

KATO, M., TOGUCHIDA, J., HONDA, K., SASAKI, M.S., IKENAGA, M., SUGMOTOT, M., YAMAGUCHI, T., KOTOURA, Y., YAMAMURO, T. & ISHIZAKI, K. (1990). Elevated frequency of a specific allele of the L-*myc* gene in male patients with bone and soft-tissue sarcomas. *Int. J. Cancer*, **45**, 47–49.

KAWASHIMA, K., SHIKAMA, H., IMOTO, K., IZAWA, M., NARUKE, T., OKABAYASHI, K. & NISHIMURA, S. (1988). Close correlation between restriction fragment length polymorphism of the L-*MYC* gene and metastasis of human lung cancer to the lymph nodes and other organs. *Proc. Natl Acad. Sci. USA*, **85**, 2353–2356.

KAWASHIMA, K., NOMURA, S., HIRAI, H., FUKUSHI, S., KARUBE, T., TAKEUCHI, K., NARUKE, T. & NISHIMURA, S. (1992). Correlation of L-*myc* RFLP with metastasis, prognosis and multiple cancer in lung-cancer patients. *Int. J. Cancer*, **50**, 557–561.

KRONTIRIS, T.G., DIMARTINO, N.A., COLB, M. & PARKINSON, D.R. (1985). Unique allelic restriction fragments of the human Ha-*ras* locus in leukocyte and tumour DNAs of cancer patients. *Nature*, **313**, 369–373.

LIDEREAU, R., MATHIEU-MAHUL, D., THEILLET, C., RENAUD, M., MAUCHAUFFE, M., GEST, J. & LARSEN, C.-J. (1985). Presence of an allelic *Eco*RI restriction fragment of the c-*mos* locus in leukocyte and tumor cell DNAs of breast cancer patients. *Proc. Natl Acad. Sci. USA*, **82**, 7068–7070.

NATIONAL CANCER INSTITUTE SPONSORED STUDY OF CLASSIFICATIONS OF NON-HODGKIN'S LYMPHOMAS (1982). Summary and description of a working formulation for clinical usage. *Cancer*, **49**, 2112–2135.

NAU, M.M., BROOKS, B.J., BATTEY, J., SAUSVILLE, E., GAZDAR, A.F., KIRSCH, I.R., MCBRIDE, O.W., BERTNESS, V., HOLLIS, G.F. & MINNA, J.D. (1985). L-*myc*, a new *myc*-related gene amplified and expressed in human small cell lung cancer. *Nature*, **318**, 69–73.

SARANATH, D., PANCHAL, R.G., NAIR, R., MEHTA, A.R., SANGHAVI, V. & DEO, M.G. (1990). Restriction fragment length polymorphism of the L-*myc* gene in oral cancer patients. *Br. J. Cancer*, **61**, 530–533.

TAMAI, S., SUGIMURA, H., CAPORASO, N.E., RESAU, J.H., TRUMP, B.J., WESTON, A. & HARIS, C.C. (1990). Restriction fragment length polymorphism analysis of the L-*myc* gene locus in a case-control study of lung cancer. *Int. J. Cancer*, **46**, 411–415.

TEFRE, T., BORRESEN, A.-L., AAMDAL, S. & BROGGER, A. (1990). Studies of the L-*myc* DNA polymorphism and relation to metastasis in Norwegian lung cancer patients. *Br. J. Cancer*, **61**, 809–812.